# 5 years DKMS Chile: approach, results and impact of the first unrelated stem cell donor center in Chile

**DOI:** 10.3389/fmed.2023.1236506

**Published:** 2023-10-12

**Authors:** Francisco Barriga, Ute V. Solloch, Anette Giani, Julia Palma, Angélica Wietstruck, Mauricio Sarmiento, Cristian Carvallo, Claudio Mosso, Pablo Ramirez, Matias Sanchez, Nicolas Rojas, Jorge Alfaro, Sebastian Saldaña, Karen Ende, Denis Flaig, Ignacia Pattillo, Alexander H. Schmidt

**Affiliations:** ^1^Fundación de Beneficencia Pública DKMS, Santiago, Chile; ^2^DKMS Group, Tübingen, Germany; ^3^Hospital Alta Complejidad Luis Calvo Mackenna, Santiago, Chile; ^4^Division de Pediatria, Red de Salud UC Christus, Santiago, Chile; ^5^Departamento de Hematologia Oncologia, Red de Salud UC Christus, Santiago, Chile; ^6^Clinica Santa Maria, Santiago, Chile; ^7^Clinica Las Condes, Santiago, Chile; ^8^Clinica Dávila, Santiago, Chile; ^9^Clinica Alemana de Santiago, Santiago, Chile; ^10^Clinica BUPA, Santiago, Chile

**Keywords:** unrelated donor center, Chile, unrelated donor recruitment, donor registry, HLA typing, stem cell transplantation

## Abstract

**Introduction:**

Allogeneic hematopoietic stem cell transplantation (allo-HSCT) is performed worldwide to treat blood cancer and other life-threatening blood disorders. As successful transplantation requires an HLA-compatible donor, unrelated donor centers and registries have been established worldwide to identify donors for patients without a family match. Ethnic minorities are underrepresented in large donor registries. Matching probabilities are higher when donors and patients share the same ethnic background, making it desirable to increase the diversity of the global donor pool by recruiting donors in new regions. Here, we report the establishment and the first 5 years of operation of the first unrelated stem cell donor center in Chile, a high-income country in South America with a population of over 19 million.

**Methods:**

We used online and in-person donor recruitment practices through patient appeals and donor drives in companies, universities, the armed forces, and public services. After confirmatory typing donors were subjected to medical work-up and cleared for donation.

**Results:**

We recruited almost 170,000 donors in 5 years. There were 1,488 requests received for confirmatory typing and donor availability checks, of which 333 resulted in medical work-up, leading to 194 stem cell collections. Products were shipped to Chile (48.5%) and abroad. Even when the COVID-19 pandemic challenged our activities, the number of donors recruited and shipped stem cell products remained steady. In Chile there was an almost 8-fold increase in unrelated donor transplantation activity from 16 procedures in 2016–2018 to 124 procedures in 2019–2021, mainly for pediatric patients following the center’s establishment. We estimate that 49.6% of Chilean patients would find at least one matched unrelated donor in the global DKMS donor pool.

**Discussion:**

Establishing a DKMS donor center in Chile has significantly increased donor availability for Chilean patients and contributed to an increase of unrelated donor stem cell transplant activity.

## 1. Introduction

Allogeneic hematopoietic stem cell transplantation (allo-HSCT) is the treatment of choice for some forms of leukemia and other life-threatening conditions (1–3). Currently, more than 50,000 allo-HSCT procedures are performed *per annum* worldwide (4). Except for haploidentical donors, allo-HSCT donors need to be HLA-matched at high resolution to avoid severe transplant-related complications, such as graft-versus-host disease (5, 6). HLA-identical siblings are preferred stem cell donors but only 20–25% of the population have such a donor (7).

Therefore, unrelated stem cell donor registries and donor centers have been established since the 1970s, primarily at the national level. The World Marrow Donor Association (WMDA) currently lists 40.6 million donors in 85 registries in 57 countries (8). These registries administer anonymous transplant-relevant data, such as HLA genotypes of registered donors, provide these data to the WMDA, and handle national and international donor searches. In contrast, donor center tasks include the following:

- Informing the public about stem cell transplantation as a curative therapy for severe blood disorders,
- Inviting them to register as potential donors,
- Collecting contact data and DNA samples for HLA typing,
- Managing the donor database and
- Transmitting anonymized data to a responsible donor registry.

Transplant centers request donors via donor registries based on search results. Donor centers communicate with the requested donors, coordinate confirmatory typing, organize medical work-up for stem cell collection from either peripheral blood or bone marrow, and follow up on the donor’s health status after donation.

Most unrelated donor transplants are performed by transplant centers in developed countries with adequate resources (9, 10). Developing countries typically have lower numbers of unrelated donor transplantations due to many factors, mainly economic, but also because of the scarce representation of regional HLA haplotypes in the global donor pool (11). The WMDA reported an unrelated donor transplant activity of 11 procedures per 10 million inhabitants in South America in 2021, compared to 93 in North America and 129 in Europe in the same year (12). In this regard, the establishment of a donor center in an ethnically diverse South American country may stimulate regional unrelated donor transplant activity (11, 13).

With 19.2 million citizens, the current Chilean population originates mainly from the admixture of European (mainly Spanish) individuals with Amerindians. 32 to 44% of Chileans have some degree of Amerindian ancestry, with the genetic composition varying by socioeconomic group (14, 15). Chile is considered a high-income country by the World Bank, with gross domestic product purchasing power parity *per capita* of US$ 27,410 in 2021 (16) and an annual health expenditure *per capita* of US$ 1,376 in 2019 (17). In 2018, Chile had nine teams performing allo-HSCT in seven centers. In that year, 155 allo-HSCT procedures were performed, representing 81 procedures per 10 million people. 84% of the transplanted products were from related donors, 14% from unrelated cord blood and only 2% from unrelated adult donors. Only one pediatric team regularly performed unrelated adult donor transplants.

DKMS is a non-profit organization based in Germany that aims to improve the situation of patients in need of stem cell transplants worldwide. For this purpose, it operates donor centers in seven countries with more than 11.7 million registered donors, the DKMS Registry, and the DKMS Life Science Lab, a high-throughput HLA typing laboratory. Since DKMS was founded in 1991, donors registered with DKMS have donated stem cells more than 105,000 times, including 7,705 times in 2022. In 2022, DKMS donors contributed 35.4% (7,705/21,767) of all unrelated stem cell collections worldwide (18). DKMS also operates an Access to Transplantation (ATT) program to overcome socioeconomic barriers to stem cell transplantation.

In this work, we report the establishment of DKMS Chile (Fundación de Beneficencia Pública DKMS), the first unrelated stem cell donor center in Chile. Our primary aim was to increase the donor pool of a population underrepresented in the global donor registry, thus improving access to transplantation for local patients.

## 2. Methods

### 2.1. Establishment of DKMS Chile

As part of its efforts to increase the odds of successful stem cell donor searches for patients globally, DKMS has screened various countries concerning their potential as a location for setting up and operating a donor center (19). Transplant-specific parameters considered in the decision process were (preferable country characteristics are given in parentheses): country-specific HLA genotypes (underrepresented in the global donor pool), number of potential stem cell donors already registered (few or none), stem cell transplant activity (existing), principal access to transplant (available at least for a substantial part of the population), position of government authorities (no reservations against establishing a donor center), legal framework (not prohibitive), and potential partners or supporters (available). Furthermore, our screening included general socioeconomic parameters as population size (large), gross domestic product/gross national income (high), health expenditures *per capita* (high), and corruption [low, operationalized by a high Corruption Perceptions Index (CPI) score (20)].

As a result of the screening, Chile turned out to be a preferable country for establishing and operating a donor center. DKMS then expressed interest in setting up a donor center in discussions with relevant individuals and organizations from Chile. Subsequently, health professionals teamed up with patient groups. They presented the need to take the offer from DKMS and establish a donor center in Chile to health authorities and relevant members of Congress. The plea was presented to the Chilean Senate in March 2016 (21) and the House of Representatives in May 2016 (22). Authorization was granted in June 2016 and preparations for the establishment of the donor center were made until its inauguration in February 2018 when the first potential donor was registered.

### 2.2. Donor recruitment

DKMS Chile recruits potential donors among people between 18 and 55 years of age who are in good health. Three main aspects interact in this endeavor.

First, it is necessary to create awareness of the need for unrelated stem cell donation and provide basic information about the topic among the general population so that donor recruitment activities can be successful. For this purpose, DKMS Chile conveys the importance of its cause via its website, social media channels, TV, radio, newspapers, and news websites. Major TV appearances, for example, were Meganoticias in October 2019, 24 Horas in February 2020, and Chilevision Noticias in September 2021. Typical communication contents include stories of patients searching for an unrelated donor, appealing donor stories, or donor-patient meetings (Supplementary Figure S1).

Second, online donor recruitment via the DKMS Chile website builds on the awareness generated in this manner. The website contains detailed information about being a registered donor, including medical requirements, as well as the steps involved in the donation process. In the online recruitment process potential donors enter personal information on the website and sign a consent form; they then receive an envelope with three swabs to obtain DNA samples of the buccal mucosa that they return to DKMS Chile. Since its inception, 201,983 kits have been requested through the website, and 87,560 returned (43.4%). To improve the return rate, alliances with pharmacies, retail stores, movie theaters, and the Santiago subway have been established to install dedicated mailboxes.

Third, the traditional donor recruitment method via so-called donor drives is often centered on a specific patient needing a transplant from an unrelated donor. In such cases, DKMS Chile, together with the family and friends of the patient, organizes a one- or two-day registration event, typically in a school, sports hall, community center, or similar location.

Donor drives, not necessarily centered around a specific patient, are also carried out at universities, companies, public services, armed forces, and other organizations where 18 to 55 year old potential donors are to be found.

Until the end of 2022, DKMS Chile has carried out 695 donor drives, including 303 in 2022. The most successful offline drives took place in Temuco and Santiago in February 2020 (6,324 donors registered in 3 days at a patient drive for a 9-year-old girl with acute leukemia) and in Talca in May 2019 (3,069 donors registered in 2 days in a patient drive for a 4-year-old girl with aplastic anemia). Owing to the COVID-19 pandemic, offline donor recruitment had to be suspended from March 2020 until December 2021. Nevertheless, a virtual drive was performed in Santiago for a 19-year-old woman with acute leukemia in September 2021, resulting in 5,360 newly registered donors.

The DNA samples of all newly registered DKMS Chile donors are genotyped for human leukocyte antigens (HLA) and other transplant-relevant parameters [e.g., ABO blood groups or CMV (cytomegalovirus) serostatus] at the DKMS Life Science Lab (Dresden, Germany) using next-generation sequencing methods (23, 24). Once the genotyping information is complete, the data are uploaded to the DKMS Registry and made available for stem cell donor searches worldwide.

### 2.3. Donor management

The main stages of the donor management process are Confirmatory Typing (CT) or Health and Availability Check (HAC), Work-Up (WU) and Follow-Up (FU). CT is mainly performed to confirm the initial HLA typing results obtained during the donor recruitment process. Donors are requested to undergo CT if their initial HLA typing results indicate that they are potential donors for a current patient. The services provided by DKMS Chile include a phone contact with the potential donor, including clarification of the continued interest to donate, a questionnaire-based health screening and the provision of donor samples for HLA typing and the analysis of infectious disease markers. Donor availability at the CT level is an important donor center quality parameter.

CT can be replaced by HAC in individual cases (25). In HAC, only the clarification of the willingness to donate and the health screening take place, and the collection of donor samples is postponed to the next process step, the donor WU.

WU includes a physical examination (PE) in the collection center followed by donor clearance, donor exclusion, or temporary deferral. The organization of the PE and, if applicable, the subsequent collection with the related travel arrangements for the donor and the coverage of all costs incurred are donor center tasks. In urgent cases, the CT or HAC step may be merged with the donor WU to reduce the time to transplant. The collection of either bone marrow (BM) or peripheral blood stem cells (PBSC) itself is no donor center responsibility but is performed by a contracted collection center. DKMS Chile currently cooperates with four collection centers that are all located in Santiago: Red de Salud UC Christus, Clinica Santa Maria, Fundación Arturo Lopez Perez, and Clinica Davila. All centers are part of recognized and certified hospital facilities accredited by the Health Ministry.

DKMS Chile conducts donor follow-up after all PBSC and BM donations to ensure the long-term safety of actual donors. The donor receives health questionnaires at defined time points after the donation (1, 6, and 12 months after donation and then yearly for 10 years) by mail and is asked to return them to DKMS Chile. Any suspicious findings are followed up in agreement with the donor.

### 2.4. Estimation of matching probabilities

Using the Hapl-o-Mat software (26, 27), we estimated Chilean 6-locus (HLA loci A, B, C, DRB1, DQB1, and DPB1) haplotype frequencies (HF) at g-group resolution (28) from 156,716 donors registered with DKMS Chile, which indicated Chile as both parents’ national origin. Haplotype frequencies <1/(2*n*) were cut, with *n* = 156,716 as the sample size. A total of 2,500 6-locus genotypes were virtually created by combining two Chilean haplotypes at random but considering the estimated HF. These 2,500 virtual genotypes represented Chilean patients in searches for 10/10 or 9/10 matched donors in the real-life DKMS Registry. This registry includes “active” donors – i.e., donors that are not flagged as temporarily unavailable at a defined time, due to health issues or a reservation for another patient, for example – from all DKMS donor centers. The investigation was conducted on January 2, 2023. The total DKMS registry size was 11,753,037 on the search date, while the DKMS Chile donor file size was 167,893. The search reports of 2,500 virtual Chilean patients were analyzed to determine matching probabilities. Non-HLA donor factors of potential relevance in real-life donor searches, such as donor age, sex, and CMV status, were not considered.

### 2.5. Survey

To investigate transplant activity in Chile since the launch of DKMS Chile compared to the years before, we surveyed allogeneic HSCT activity from 2016 to 2021 among four pediatric and seven adult transplant teams in Chile that account for most of the transplant activity in the country. All centers were located in Santiago. All donor types were included in the activity survey: unrelated cord blood units (UCB), matched related donors (MRD), haploidentical family donors (Haplo), and matched or mismatched unrelated donors (URD).

## 3. Results

### 3.1. Donor recruitment and donor file composition

**Figure 1:**
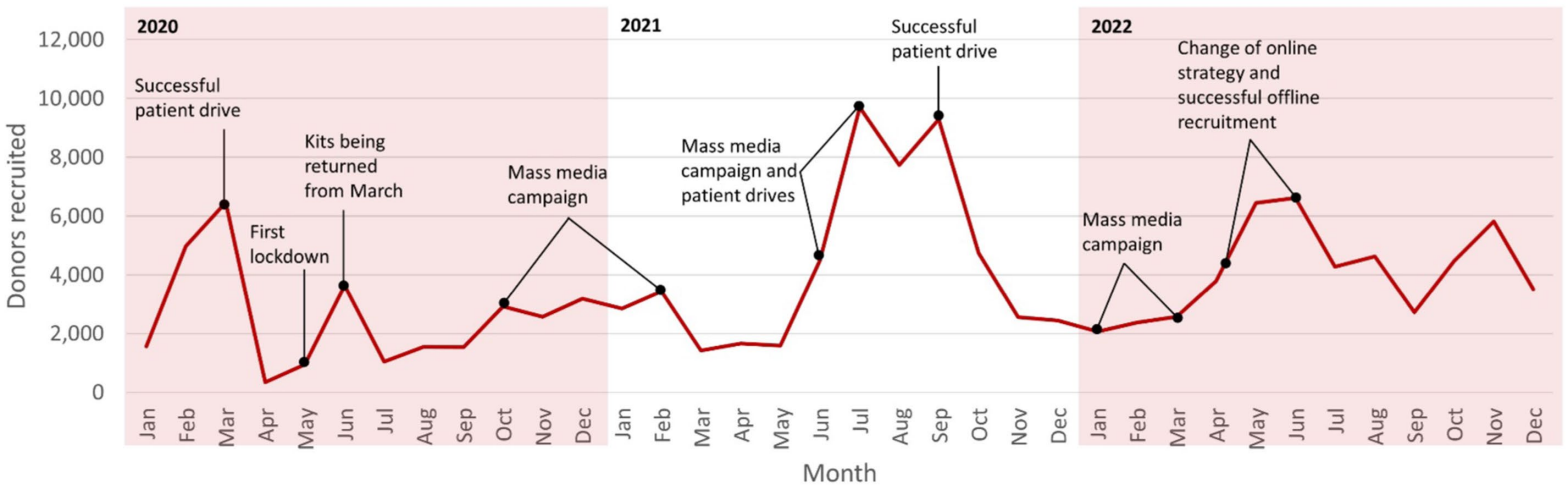
Fluctuations of the donor recruitment success of DKMS Chile over time (2020–2022).

Since the foundation of DKMS Chile in February 2018, 169,899 donors have been recruited until December 31, 2022, including 83,340 via offline donor drives (695 patient drives and 199 organization drives at universities, companies, public services, or armed forces) and 86,559 via online donor recruitment. The respective 2022 figures were 49,250 newly recruited donors, including 29,532 in offline donor drives and 19,718 online. Donor recruitment success was subject to significant fluctuations over time (Figure 1). Since the foundation of DKMS Chile, two major societal incidents have considerably impacted donor recruitment activities and their success: the deep political crisis and social uprising in Chile in October 2019 and the following months, and the COVID-19 pandemic since the beginning of 2020.

**Figure 2:**
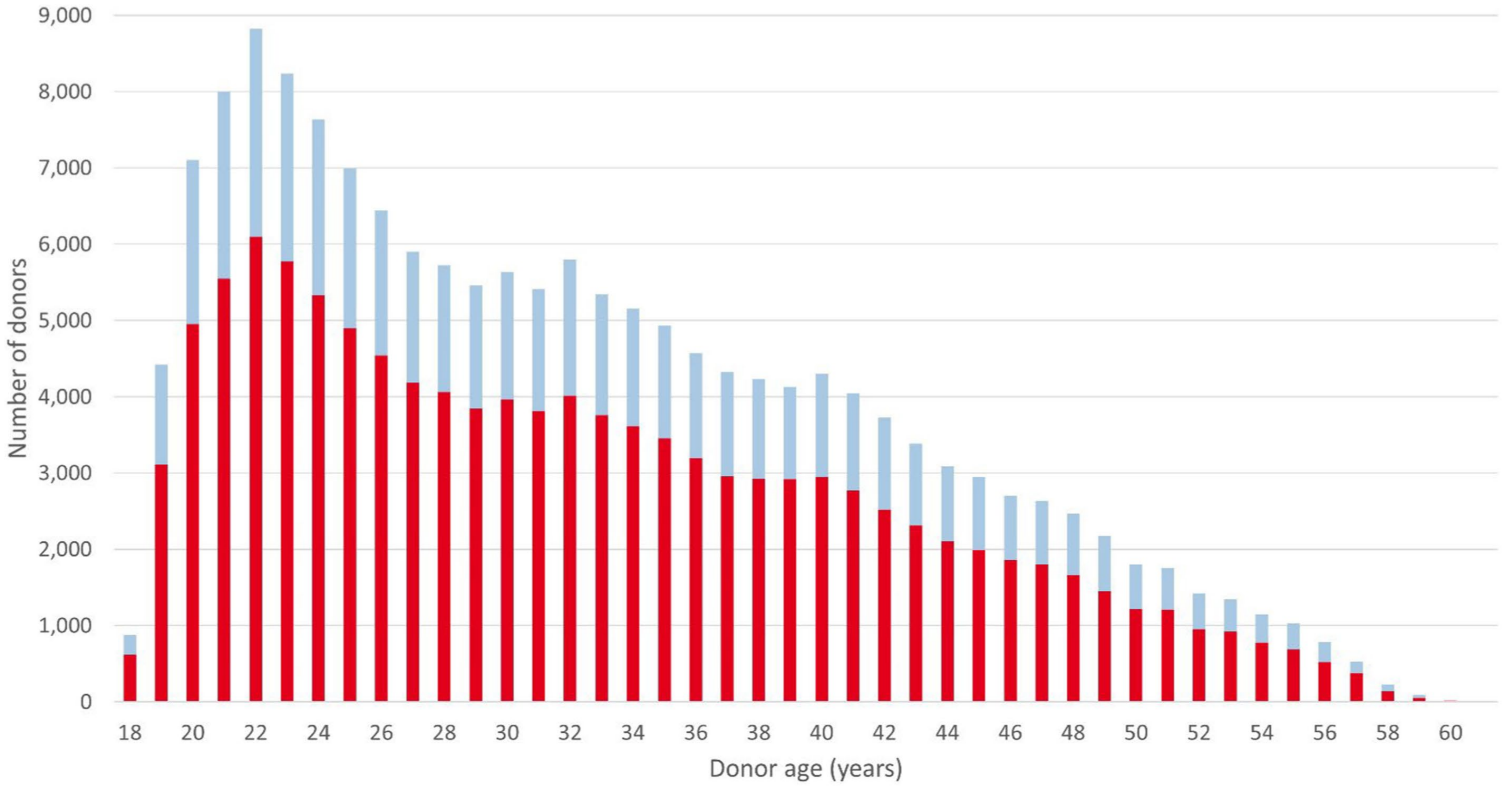
Age and sex distribution of donors registered with DKMS Chile. Data retrieval date: December 31, 2022. Red: female; blue: male.

167,346 of the recruited donors were active on December 31, 2022. Of these 167,346 donors, 116,159 (69.4%) were female and 51,187 (30.6%) were male. The average (median) donor age was 32.4 (29) years. The number of male donors aged 25 years or younger was 15,797, representing 9.4% of the total DKMS Chile donor file (Figure 2). Young male donors are of specific relevance to the patient benefits that a donor center can generate, as these donors are preferably selected by transplant physicians (30, 31).

Regarding ancestry, unsurprisingly, the vast majority of registered donors (156,716 out of 167,346; 93.6%) indicated that both parents were of Chilean descent, followed by donors with parents of Venezuelan (1,904, 1.1%), Colombian (894, 0.5%), and Argentinean (633, 0.4%) origin. Among donors of Chilean descent, 124,306 (79.3%) reported both parents to be of non-indigenous ancestry, while 2,501 (1.6%) reported both parents to be of Mapuche origin, and 5,913 (3.8%) reported one parent to be of non-indigenous and Mapuche descent (Supplementary Tables S1, S2).

### 3.2. Donor requests and stem cell donations

**Figure 3:**
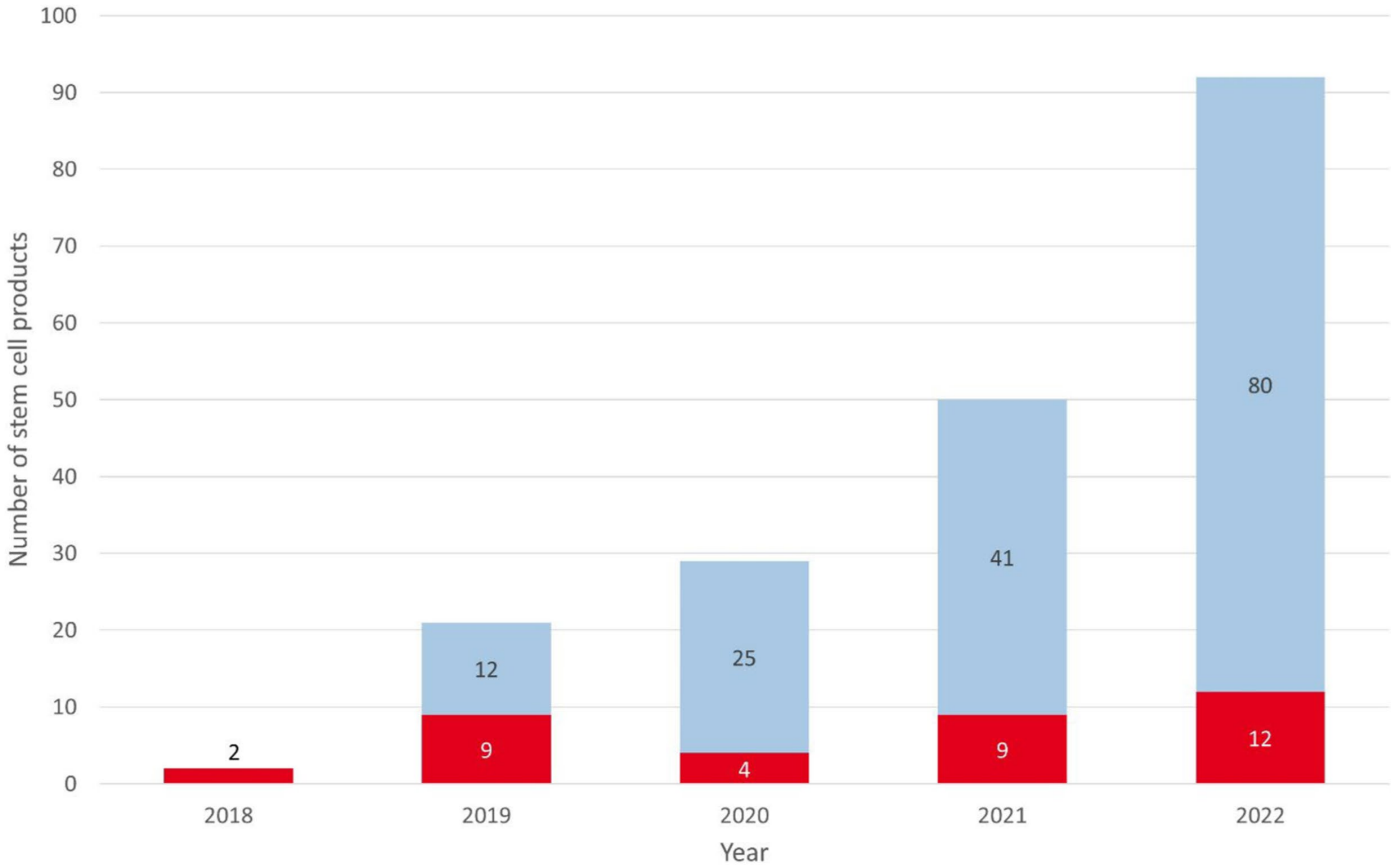
Number of stem cell products provided by DKMS Chile from 2018 to 2022. Red: BM; blue: PBSC.

**Figure 4:**
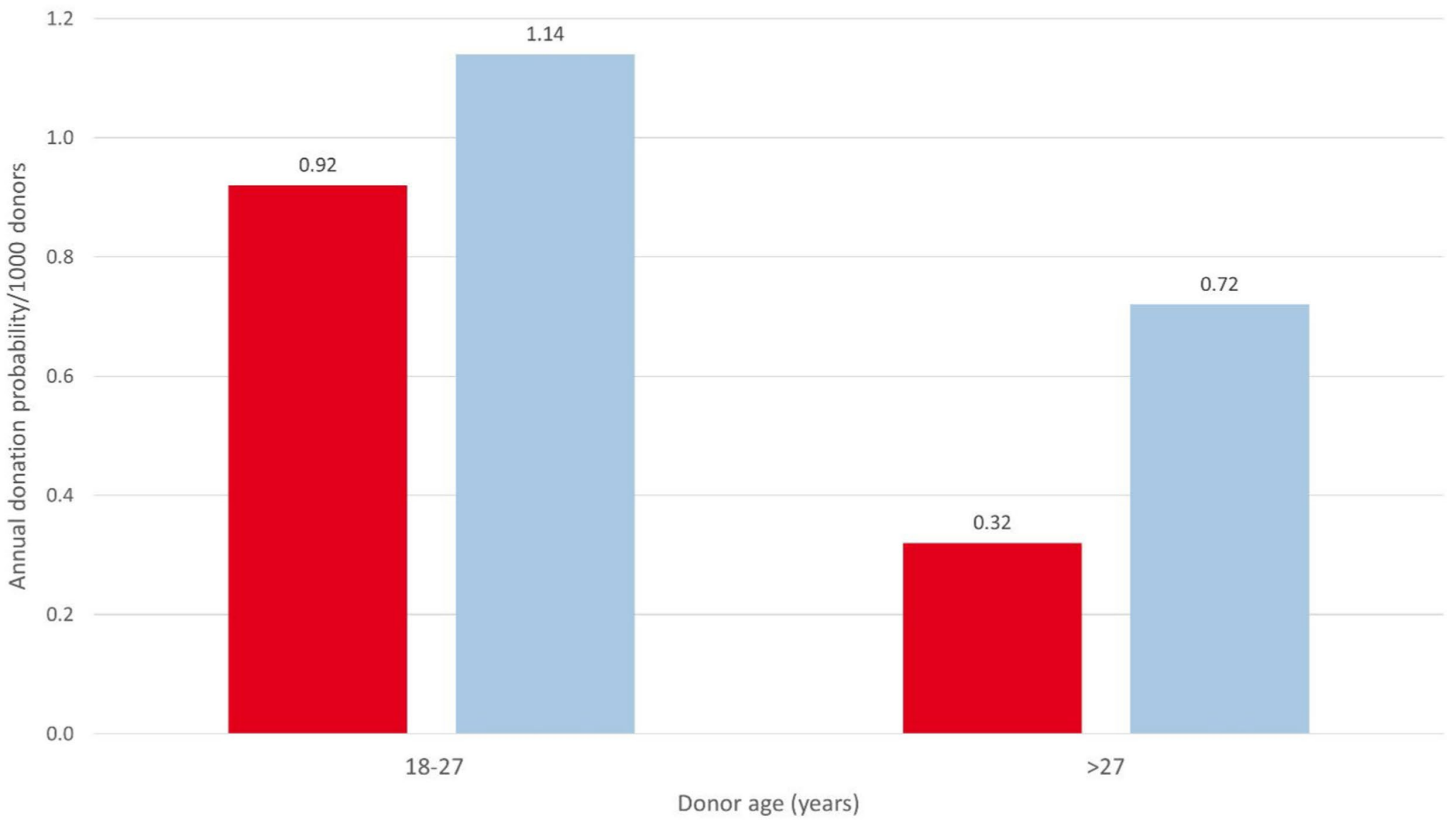
Annual donation probabilities of DKMS Chile donors by donor sex and age group. Red: female; blue: male.

From February 2018 to December 2022, DKMS Chile received 1,366 CT and 122 HAC requests. CT requests originated mainly from Chile (33.1%), the United States (26.1%), and Argentina (10.1%; Supplementary Table S3). Donor availability at CT level was 63.2% (396/627) in 2022. The corresponding values for other DKMS donor centers were between 29.9% (DKMS-BMST India) and 75.0% (DKMS Germany) in that year. The main reasons for unavailability of DKMS Chilean donors were lack of interest (18.0%) and temporal unavailability (10.0%). CT and HAC activities led to 333 donor WU requests, resulting in 194 stem cell donations (158 PBSC and 36 BM), including 92 donations in 2022 (80 PBSC and 12 BM). Stem cell products were shipped to Chile (94/48.5%), the United States (33/17.0%), Spain (17/8.8%), and 16 other countries (Supplementary Table S4). 56.2% (109/194) of all products went to patients from Latin American countries. Of the products for Chilean patients, 79 (84.0%) were for pediatric patients (up to 18 years) and 15 (16.0%) for adult patients. Diagnoses included acute leukemia (73.1%), immunodeficiencies (11.8%), severe aplastic anemia (8.6%), other hematologic malignancies (3.2%), and other non-malignant diseases (3.2%). Donation figures show a clear growth trend, with 84% growth (from 50 to 92) from 2021 to 2022 (Figure 3). The usage rate (products collected per 1,000 registered donors) of the DKMS Chile donor file was 0.41 in 2021 and 0.54 in 2022, compared to the usage rate of the global stem cell donor pool of 0.53 in 2021 (12). Based on the 2022 donation figures, the annual usage rate of DKMS Chilean donors by age ranged from 0.32 (female donors, >27 years) to 1.14 (male donors, 18–27 years) collections per 1,000 donors (Figure 4).

### 3.3. Matching probabilities

In searches for 2,500 virtual Chilean patients, at least one 10/10 matched donor could be found in the real-life DKMS Registry (*n* = 11,753,217) in 49.6% of cases. In the much smaller subset of DKMS Chilean donors (*n* = 167,893, 1.4% of the total DKMS Registry size), 35.8% of all searches still resulted in the identification of a fully (10/10) matched donor. Interestingly, in 21.5% of the searches, 10/10 matched donors were only found in DKMS Chile. Therefore, the odds for Chilean patients to find a 10/10 matched donor in the DKMS Registry would drop substantially from 49.6 to 28.1% without donors from DKMS Chile.

In 50.4% (1,260/2,500) of the searches, no 10/10 matched donor could be found in the entire DKMS Registry. In 69.2% of these 1,260 searches, we identified at least one 9/10 matched donor, so that at least one ≥9/10 matched donor was found for 84.5% of all virtual patients. This success rate would decrease to 62.6% without the DKMS Chile donors, thus further confirming the relevance of domestic donor recruitment for Chilean patients.

### 3.4. Development of allogeneic transplant activity

**Figure 5:**
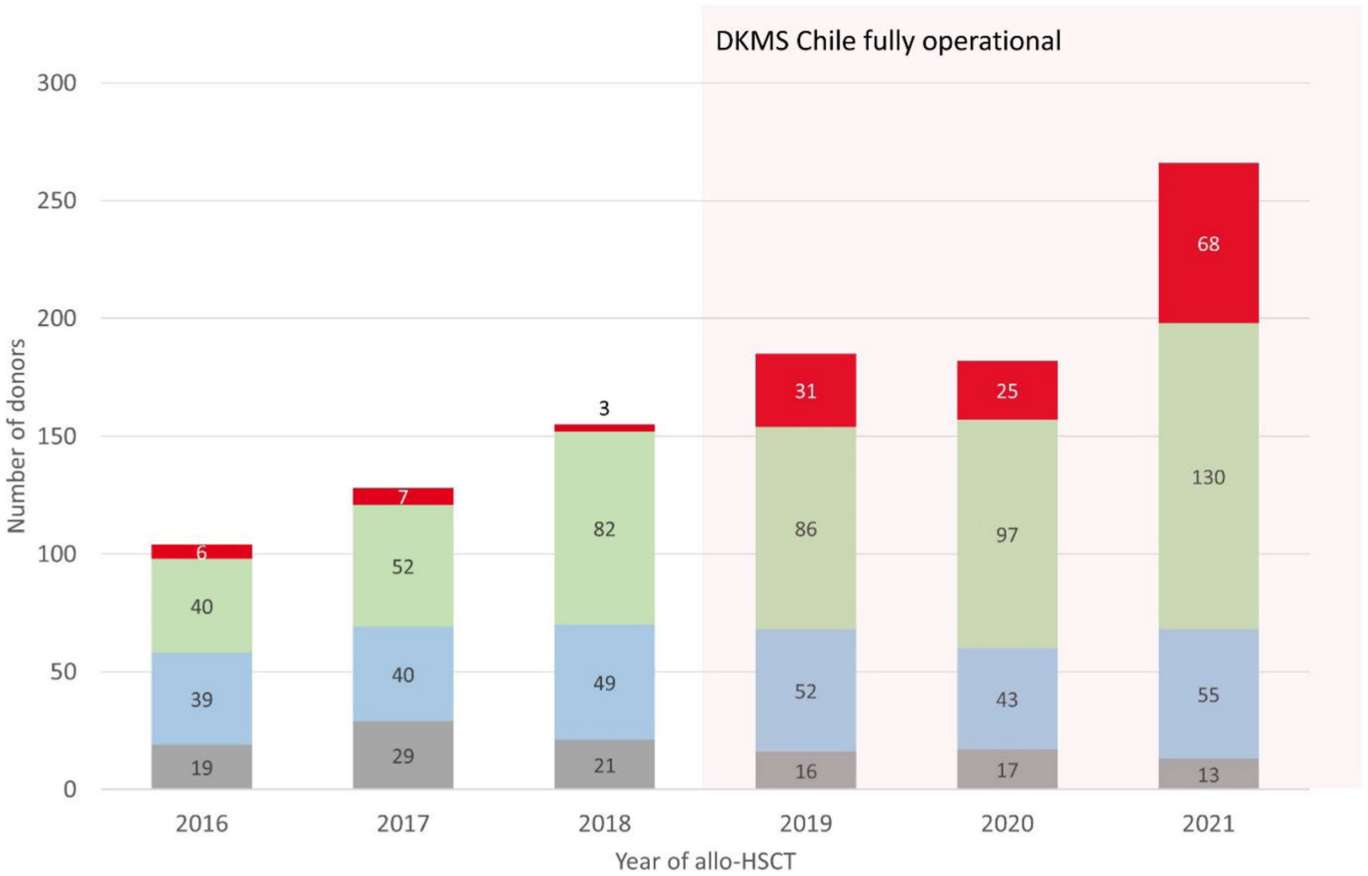
Development of allo-HSCT numbers in Chile by donor type from 2016 to 2021. UCB, umbilical cord blood, grey; MRD, matched related donor, blue; Haplo, haploidentical donor, green; URD, unrelated donor, red.

In an allo-HSCT activity survey, Chilean teams reported 1,020 procedures from 2016 to 2021. We compared the activity between 2016 and 2018, and 2019 and 2021 after DKMS Chile became fully operational. Allogeneic transplant activity increased by 63.6%, from 387 procedures in the first period to 633 in the second. URD transplants showed the most significant increase, from 16 in 2016–2018 to 124 in 2019–2021, an almost 8-fold increase (Figure 5).

Children represented 84.7% of recipients (105/124). Pediatric allo-HSCT from all donor types in 2021 corresponded to a ratio of 26 transplants per one million children per year (100 transplants per 3.8 million children). In 2020, the transplant activity in Chile was 17.6 transplants per one million children, which compares with the United States HRSA report of 19.6 allogeneic transplants per one million children (1,390 transplants per 70.9 million children) (29). In that same year adult transplant teams performed 114 allo-HSCTs, corresponding to 6.0 transplants per one million Chilean adults, compared to 22.5 transplants per one million adults reported to HRSA. Only one team transplanted adult patients with stem cell products from unrelated donors in 2020.

**Figure 6:**
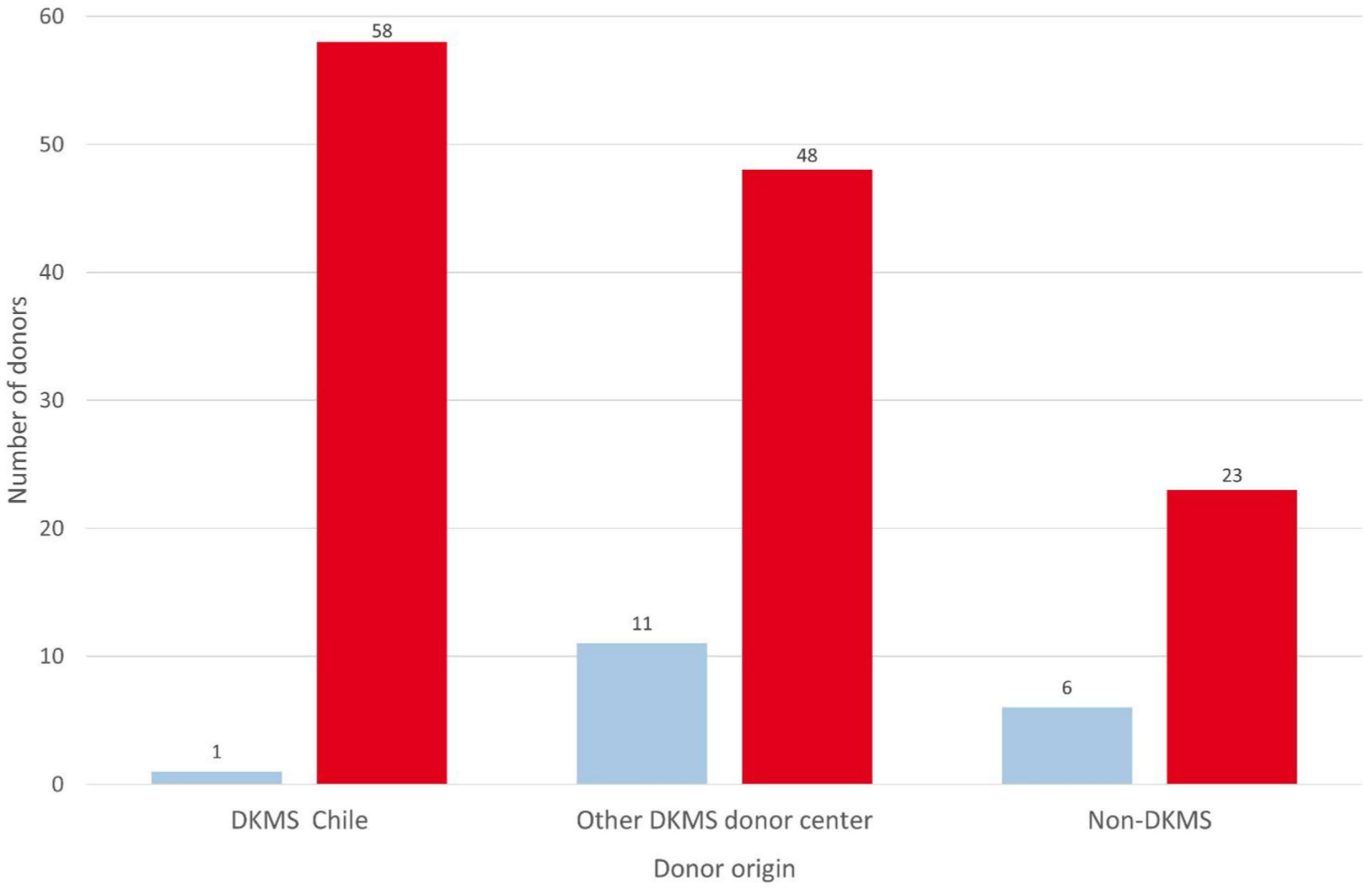
Donor origin of URD stem cell products for Chilean patients. Blue: 2016–2018; red: 2019–2021.

According to WMDA and DKMS internal data, in the period from 2019 to 2021, 58 products originated from DKMS Chile (45.0%), 48 from other DKMS donor centers (37.2%), and 23 from non-DKMS donor centers (17.8%) (Figure 6). The numbers reported by the WMDA and the survey do not perfectly match for URD and CBU products, since WMDA is reporting shipped products and the survey reports transplanted products. Specifically, during the COVID-pandemic not all cryopreserved URD products have been infused (32).

## 4. Discussion

We report the achievements of the first 5 years of DKMS Chile, the first stem cell donor center in Chile. We currently administer close to 170,000 registered donors and provided 194 stem cell products to patients in need, including 94 patients from Chile, until the end of 2022. Despite restraints related to social upheaval and the COVID-19 pandemic, donor recruitment and management activities have proceeded successfully.

The positive development of DKMS Chile strengthens our conviction that the country selection criteria given in the method section are reasonable. We are grateful for the support by the political and health authorities.

Our calculations regarding matching probabilities show that DKMS Chile, although it currently represents only about 0.4% of the global donor pool, already benefits Chilean patients by increasing the odds of finding a 10/10 or 9/10 matched unrelated stem cell donor substantially. This observation confirms previous results, indicating the importance of donor recruitment efforts in the same population from which the patients originate (33–35).

The survey results show a significant increase in allo-HSCT in Chile in a close temporal relation to the launch of DKMS Chile, especially in pediatric patients transplanted with unrelated donors. Nevertheless, it cannot be assumed that DKMS Chile alone caused this positive development. This achievement also included many transplant center factors, including increased bed capacity, collaboration between public and private health systems that increased patient admission to transplant, and improved funding. The number of URD transplantations has increased due to center preferences and donor availability. The latter issue suggests that DKMS Chile has played a considerable role in this multifactorial positive development. During the period surveyed, allogeneic transplant activity for adult patients also increased in the country, from 244 (2016–2018) to 397 (2019–2021) procedures. Adult transplant teams in Chile prefer haploidentical related donors over unrelated donors for patients who lack matched sibling donors. Nevertheless, since DKMS was established in the country, some centers have started performing unrelated donor transplantation for adult patients (18 patients in 2020–2021).

Interestingly, the number and proportion of products going to foreign patients increased from 17 (34%) in 2021 to 57 (62%) in 2022. Six products went to other Latin American countries and 23 to the United States in 2022. We see the growing export rate as evidence that DKMS Chile is increasingly seen by the international community as an “established” donor center. Stem cell product exports are also important for reasons of financial self-sustainability. While DKMS Chile provides products for domestic patients at cost price or below, products for non-domestic patients include a margin and thus help to reduce funding demands of DKMS Chile in the long term.

DKMS Chile is in a special situation as it is part of the DKMS network of donor centers with a total of more than 11 million registered donors. This also includes access to a wealth of experience, established processes, and IT solutions. Basically, we follow a copy-paste approach when possible when establishing a new donor center and seeking new, innovative solutions where required. Establishing a donor center outside such a network is certainly much more difficult and involves higher risks. DKMS can and is willing to provide support in such cases through knowledge sharing and appropriate training.

## Data availability statement

The original contributions presented in the study are included in the article/Supplementary material, further inquiries can be directed to the corresponding author.

## Author contributions

FB: manuscript design, data analysis, writing, and transplant activity survey. AG, US, and DF: data capturing and analysis, matching probabilities analysis, and manuscript review. JP, AW, MauS, CC, CM, MatS, PR, NR, and JA: transplant survey data capturing and manuscript review. KE and AS: manuscript writing and review. All authors contributed to the article and approved the submitted version.
