# Supplementary figures and images for: 5 years DKMS Chile: approach, results and impact of the first unrelated stem cell donor center in Chile
Source: Front Med (Lausanne). 2023 Oct 12;10:1236506. doi: 10.3389/fmed.2023.1236506 (PMC10601464; doi:10.3389/fmed.2023.1236506)

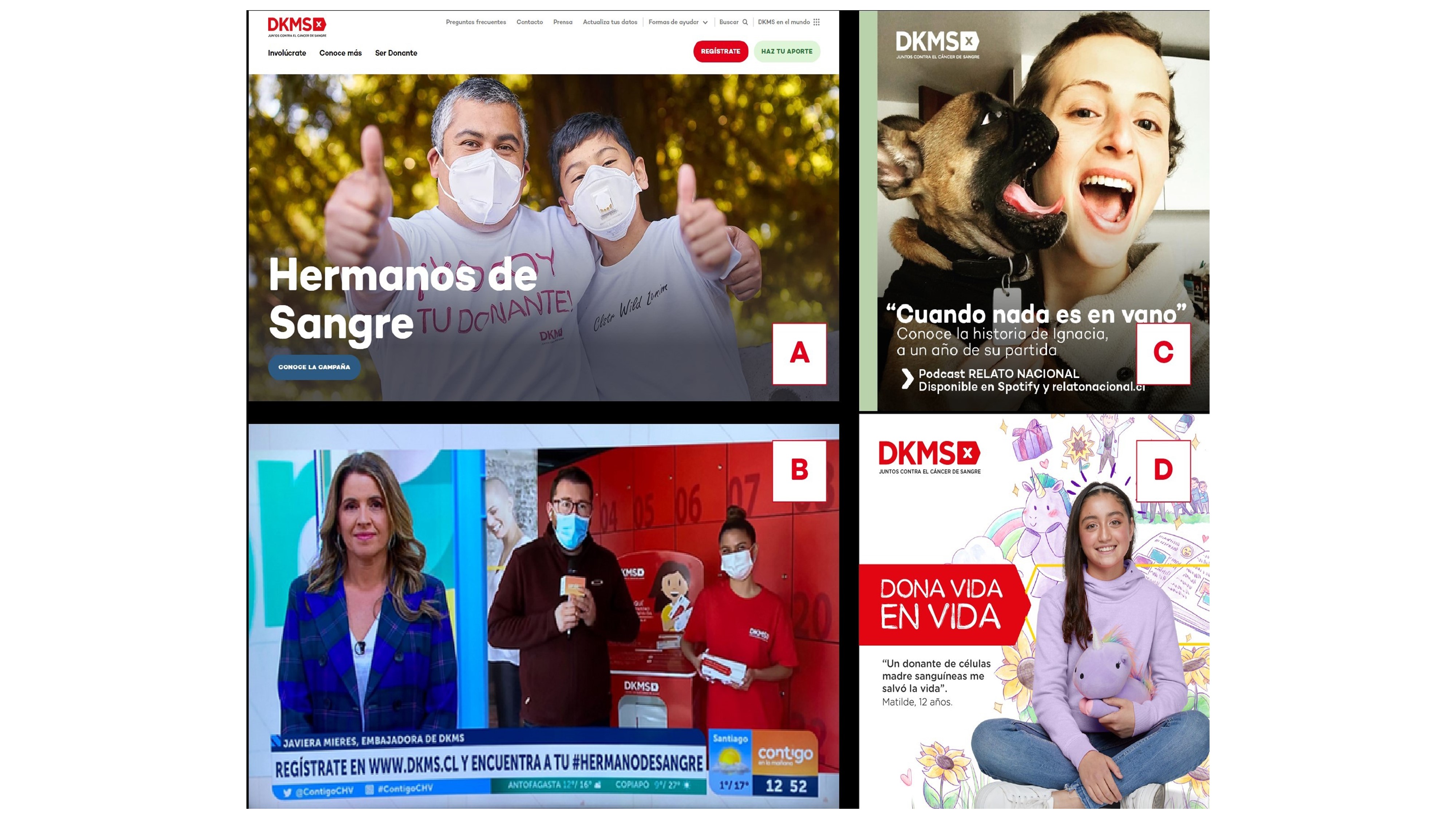

Supplement: Supplementary file 2 [file Image_1.jpeg]
